# A Class of Promising Acaricidal Tetrahydroisoquinoline Derivatives: Synthesis, Biological Evaluation and Structure-Activity Relationships

**DOI:** 10.3390/molecules19068051

**Published:** 2014-06-16

**Authors:** Rui Yang, Qiao Ruan, Bing-Yu Zhang, Zuo-Lue Zheng, Fang Miao, Le Zhou, Hui-Ling Geng

**Affiliations:** 1 College of Science, Northwest A&F University; Yangling, Shaanxi 712100, China; E-Mails: yrer01018@126.com (R.Y.); qiaoruanchem@gmail.com (Q.R.); bingyuzhang607@gmail.com (B.-Y.Z.); zhengzuolue@gmail.com (Z.-L.Z.); 2 College of Life Science, Northwest A&F University; Yangling, Shaanxi 712100, China; E-Mail: miaofangmf@163.com

**Keywords:** tetrahydroisoquinoline, acaricidal activity, *Psoroptes cuniculi*, structure-activity relationship

## Abstract

As part of our continuing research on isoquinoline acaricidal drugs, this paper reports the preparation of a series of the 2-aryl-1-cyano-1,2,3,4-tetrahydroisoquinolines with various substituents on the *N*-phenyl ring, their *in vitro* acaricidal activities against *Psoroptes cuniculi*, a mange mite, and discusses their SAR as well. The structures of all compounds, including 12 new ones, were elucidated by analysis of UV, IR, NMR, ESI-MS, HR-MS spectra and X-ray diffraction experiments. All target compounds showed varying degrees of activity at 0.4 mg/mL. Compound **1** showed the strongest activity, with a 50% lethal concentration value (LC_50_) of 0.2421 μg/mL and 50% lethal time value (LT_50_) of 7.79 h, comparable to the standard drug ivermectin (LC_50_ = 0.2474 μg/mL; LT_50_ = 20.9 h). The SAR showed that the substitution pattern on the *N*-aromatic ring exerted a significant effect on the activity. The substituents 2'-F, 3'-F, 2'-Cl, 2'-Br and 2'-CF_3_ remarkably enhanced the activity. Generally, for the isomers with the same substituents at different positions, the order of the activity was *ortho* > *meta* > *para*. It was concluded that the target compounds represent a class of novel promising candidates or lead compounds for the development of new tetrahydroisoquinoline acaricidal agents.

## 1. Introduction

Mites are ectoparasites that can cause a chronic skin disease, acariasis, which occurs widely in animals and human. *Psoroptes cuniculi* is an animal mite that mainly infects rabbits, goats, horses and sheep [1]. It causes intense pruritus in animals, with the formation of crusts and scabs, reduction of weight gain, and even death [2,3], therefore, this mite species causes serious economic losses for the animal industry.

Therapy and control of both human scabies and animal mange have been based mainly on the use of effective drugs and chemicals. Among them, ivermectin is the most clinically effective acaricide. But as it is being used increasingly, drug-resistance of mites has developed [4,5], which often leads to drug-treatment failures, recrudescence or reinfection [6]. In addition, concerns over the environmental damage [7,8] and the toxicity of many acaricides limit their application and reduces the number of safe effective products available. These problems have prompted us to dedicate great efforts to develop new effective and safe acaricides from active natural products.

**Figure 1:**
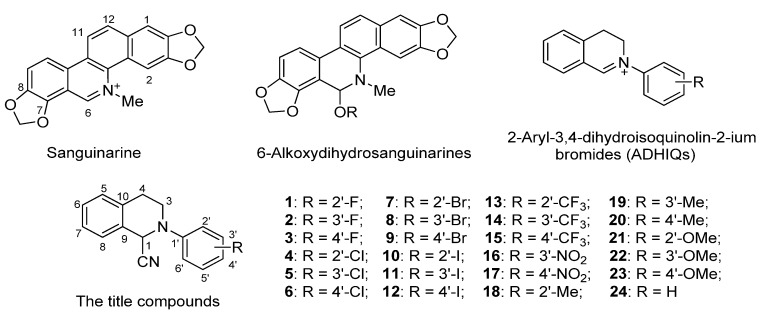
Structures of sanguinarine, 6-alkoxy dihydrosanguinarines, 2-aryl-3,4-dihydroisoquinolin-2-iums and the title compounds.

Our previous research documented that 6-alkoxydihydrosanguinarines (Figure 1), derivatives of isoquinoline alkaloid sanguinarine, possessed significant acaricidal activity *in vitro* against *P. cuniculi*, compared with the commercial acaricide ivermectin [9]. Thereafter, in order to develop more effective sanguinarine-like acaricidal drugs, we designed a class of structurally simple analogues of sanguinarine, *i.e.*, 2-aryl-3,4-dihydroisoquinolin-2-iums (ADHIQs, Figure 1), by imitating the structural characteristics of sanguinarine, and found that most of the ADHIQs had higher activity than the 6-alkoxydihydrosanguinarines and ivermectin [10].

However, both the 6-alkoxydihydrosanguinarines and ADHIQs are not completely satisfactory because of their drawbacks of chemical instability or high chemical reactivity [11,12,13,14,15,16], which makes the drug molecules easily loss the bioactivity under physiological conditions. Thus, it is essential to improve the bioavailability of ADHIQs to develop practical isoquinoline acaricides.

The purpose of our current research is to examine *in vitro* the acaricidal activity of a series of the iminium moiety-modified derivatives of ADHIQs, *i.e.*, 1-cyano-2-aryl-1,2,3,4-tetrahydroisoquinolines (CATHIQs, Figure 1), against *P. cuniculi* and disclose their SAR. We expected that the stability and diversity of the target compounds as well as their structural similarity to ADHIQs could provide us with new acaricidal compounds with high stability. As far as we know, this is the first report on acaricidal activity of the target compounds.

## 2. Results and Discussion

### 2.1. Chemistry

**Scheme 1:**
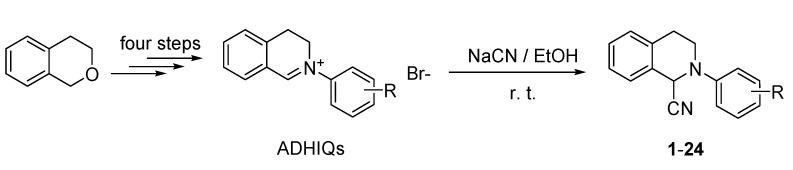
Synthesis of the title compounds **1**–**24**.

The chemical synthesis of the target compounds is outlined in Scheme 1. According to our previously reported method [10], the intermediate 2-aryl-3,4-dihydroisoquinolin-2-ium bromides were prepared in four steps by using isochromane as a starting material. At room temperature, 24 target compounds including 12 new ones (compounds **2**, **7**, **8**, **10**–**18**) were obtained by the reaction of sodium cyanide with the corresponding 2-aryl-3,4-dihydroisoquinolin-2-ium bromides in 64%–98% yields using 75% ethanol in water (v/v) as solvent. It was noteworthy that the reaction of 2-(2-nitrophenyl)-3,4-dihydroisoquinolin-2-ium bromide with sodium cyanide under the same condition did not give the expected compound.

The structures of all target compounds were elucidated by spectroscopic analysis. Each of the compounds showed characteristic ion peaks at *m*/*z* [M−CN], [M+H], [M+Na] in the positive electrospray ionization MS spectrum, and a strong absorption (C≡N) in the range of 2219 to 2226 cm^−1^ in the IR spectrum. In the ^1^H- and ^13^C-NMR spectra, all compounds revealed singlet signals in the range of *δ* 5.13 to 5.60 ppm assigned to H-1 and one signal in the range of *δ* 51.3 to 56.2 ppm due to C-1. An exception was **17**, with absorption signals at 2,361 cm^−1^ (C≡N) in the IR spectrum, and *δ*_H-1_ 5.79 and *δ*_C-1_ 61.4 ppm in its NMR spectra. In addition, it was noteworthy that the *δ*_H-1_ signals of **18** and **21** in their ^1^H-NMR appeared at 5.06 ppm in the higher field and 5.74 ppm [17] in the lower field, respectively. The structures of all new compounds were further confirmed by HR-MS data.

**Figure 2:**
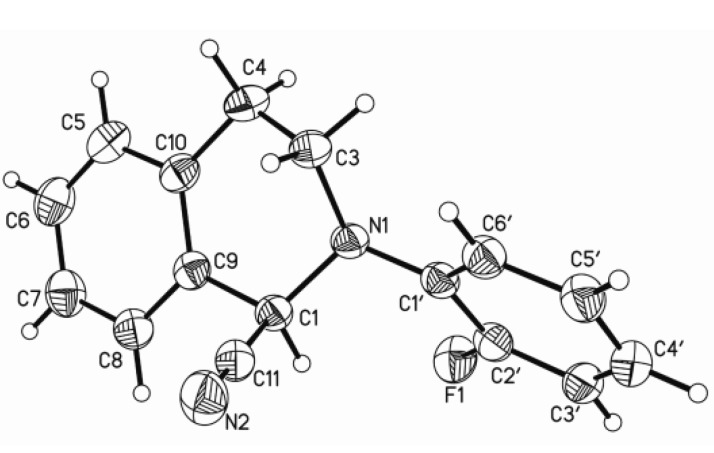
Crystal structure of compound **1**.

The structure of compound **1** was further supported by a single-crystal X-ray diffraction experiment (Figure 2). Its crystallographic data revealed that the dihedral angle between two aromatic rings was 52.041(42)°. In the six-membered heterocyclic ring, C7/C6/C1/C9 was approximately coplanar, and N1 and C8 deviated from it by 0.3962 Å and 0.3792 Å, respectively. In addition, there existed a 2.32 Å intra-annular hydrogen bond between H9 and F.

### 2.2. In Vitro Acaricidal Activity and Structure-Activity Relationships

**Figure 3:**
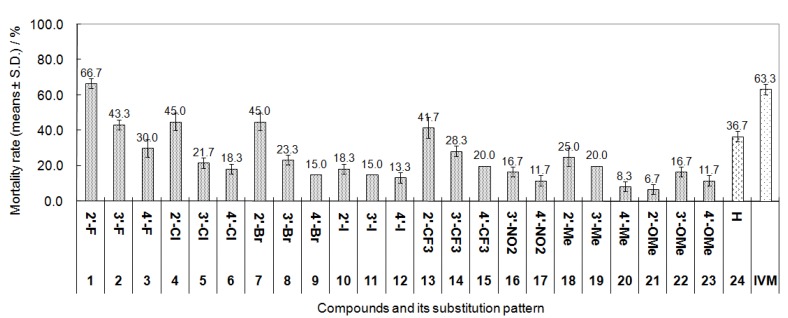
The acaricidal activities of compounds **1**–**24** against *P. Cuniculi* at 0.4 mg/mL.

**Note**: IVM refers to ivermectin, a standard acaricide, as positive drug control.

The *In vitro* acaricidal activity of the compounds **1**–**24** was tested according to our previous method [10]. Ivermectin, a standard acaricide, was selected as the positive drug control. The results are shown in Figure 3.

All compounds showed different degrees of activity at 0.4 mg/mL. Among them, **1** displayed the highest activity, with an average mortality rate of 66.7%, slightly higher than that of ivermectin (63.3%). Compounds **2**, **4**, **7**, **13** and **24** revealed moderate activities, with average mortality rates of 36.7% to 45.0% and the other compounds exhibited lower activities with the average mortality rates of 6.7% to 30.0%.

Comparison of the activity of **24** without substituents on the *N*-benzene ring with that of **1**–**23** showed that the characteristics and position of the substituent on the *N*-phenyl ring had a significant effect on the activity. The general trend was that the introduction of 2'-F (**1**), 3'-F (**2**), 2'-Cl (**4**), 2'-Br (**7**) or 2'-CF_3_ (**13**) on the *N*-aromatic ring led to an obvious improvement of the activity. On the contrary, the introduction of an iodine atom (compounds **10**–**12**), methyl (compounds **18**–**20**), methoxy (compounds **21**–**23**) or nitro group (compounds **16**–**17**) to any position of *N*-aromatic ring caused the reduction of the activity to varying degrees.

For various isomers with the same substitution, it was clearly observed that the position of the substituent had a significant effect on their activity. Generally, except for methoxy- and nitro-substituted compounds (compounds **16**–**17**, **21**–**23**), the order of the activity of each set of isomers was 2'-substituted isomer > 3'-substituted isomer > 4'-substituted isomer. For example, the mortality rates of 2'-, 3'- and 4'-F isomers **1**–**3** were 66.7%, 44.3% and 30.0%, respectively. Therefore, for most of the substituents, the 2'-position was considered as an optimal modifiable site for the improvement of the activity.

Furthermore, comparing the activities of the compounds with various substituents at the same position, it was found that the impact of various substituents on the activity was totally different. In most cases, the intensity order of the substituents’ influence on the activity was as follows: fluorine atom (compounds **1**–**3**) > chlorine atom (compounds **4**–**6**) ≈ bromine atom (compounds **7**–**9**) ≈ trifluoromethyl (compounds **13**–**15**) > iodine atom (compounds **10**–**12**) ≈ methyl (compounds **18**–**20**) > methoxy (compounds **21**–**23**) ≈ nitro (compounds **16**–**17**).

**Figure 4:**
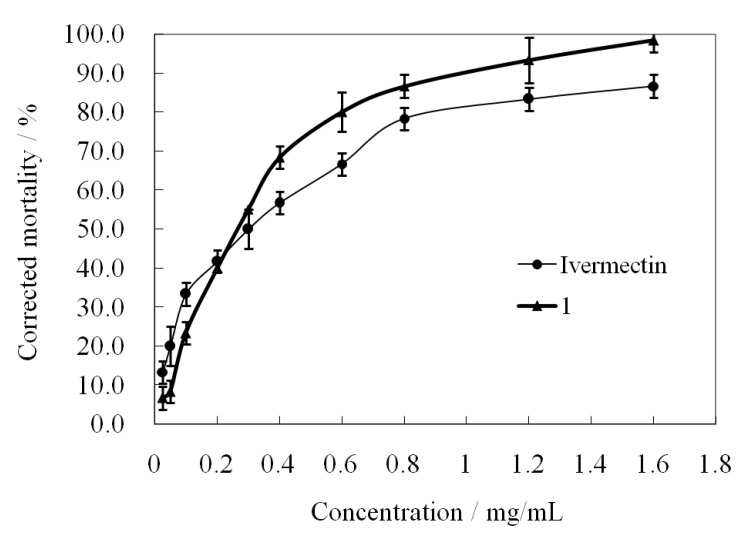
The acaricidal activities of **1** and ivermectin at various concentrations at 24 h.

To explore in more detail the acaricidal potency of all compounds, compound **1** with the highest activity was subjected to an acaricidal toxicity assay using ivermectin as a standard drug control. The acaricidal activities against *P. cuniculi* caused by the treatment with various concentrations of **1** and ivermectin at 24 h post-treatment are shown in Figure 4. Their toxicity regression equations for concentration effects are listed in Table 1.

**Table 1:** Toxicity regression equations for concentration effect and LC_50_ values (mg/L) of **1** against *P. cuniculi* (24 h).

Compound	Toxicity Equation *^a^*	R^2^	LC_50_/mg/mL (mM)	95%CI of LC_50_ *^b^*	Linear Scope/μg/mL
**1**	*y* = 2.1170*x* − 0.0469	0.9947	0.2421 (0.960)	0.2354–0.2490	0.1000–1.200
**Ivermectin**	*y* = 1.3165*x* + 1.8491	0.9804	0.2474 (0.283)	0.1979–0.3102	0.0500–1.600

*^a^* y: The probability of the mortality; x: log [concentration (μg/mL)]; *^b^* 95% CI: lower and upper values of the confidence interval of LC_50_ (mg/mL) at 95% probability.

The results of Figure 4 proved that the activities of **1** and ivermectin were enhanced with the increase of their concentration. It was worth noting that there was a cross point between the two curves at a concentration of *ca.* 0.2 mg/mL, indicating that **1** was more effective than ivermectin at more than *ca.* 0.2 mg/mL. A similar case was also observed for 6-methoxydihydrosanguinarine [9]. Statistical analysis showed that both **1** and ivermectin had a significant linear correlation between the probability of mortality rates and log[concentration] values (*R*^2^ > 0.98) (Table 1). Although **1** and ivermectin showed approximately identical median lethal concentration values (LD_50_ = 0.2421, 0.2474 mg/mL), the toxicity regression equation of **1** had a much larger slope ratio value (*k* = 2.1170) than that of ivermectin (*k* = 1.3165), indicating that the activity of **1** had more sensitive concentration-dependence than that of ivermectin.

**Figure 5:**
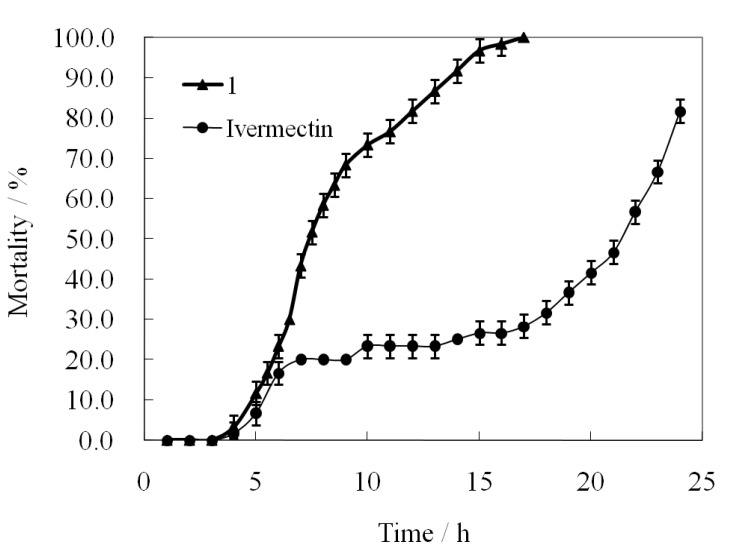
The acaricidal activities of **1** and ivermectin at 3.0 mg/mL at various post-treatment times.

The acaricidal activities caused by treatment of the same concentration of **1** and ivermectin (3.0 mg/mL) at various treatment times are shown in Figure 5 and their toxicity regression equations for time effect are listed in Table 2. Like in the case of the concentration effect, the acaricidal activities of the two compounds were heightened with the prolongation of the treatment time (Figure 5).

**Table 2:** Toxicity regression equations for treatment time effect and LT_50_ values of **1** against *P. cuniculi* (3.0 mg/mL).

Compound	Toxicity Equation *^a^*	R^2^	LT_50_/h	95%CI*^ b^* of LT_50_	Linear Scope/h
**1**	*y* = 5.424*x* + 0.1657	0.9742	7.79	7.59–8.03	5.5–14.0
**Ivermectin**	*y* = 7.3816*x* – 4.7427	0.9491	20.89	20.75–20.93	17.0–23.0

*^a^* y: The probability of the inhibition rate; x: log [treat time (h)]; *^b^* 95% CI: lower and upper values of the confidence interval of LT_50_ (h) at 95% probability.

Meanwhile, we also observed some distinct differences between the two curves. At every treatment timepoint, compound **1** nearly showed higher activity than ivermectin. After 4 h post-treatment, the mortality rates caused by **1** began to quickly increase as the time went on, and finally reached 100% at 17 h. Unlike the case of **1**, the activity of ivermectin showed one slow increase in the 6 h to 17 h range. Before 17 h post-treatment, ivermectin gave lower mite mortality rates (<30%). Even by 24 h post-treatment, ivermectin did not cause the death of all mites. Statistical analysis showed that **1** had a significant linear correlation between the mortality rate probability and log[treatment time] values (*R*^2^ = 0.9742) (Table 2) in the range of 5.5 h to 14 h, and its median lethal times (LT_50_ = 7.79 h) was much smaller than that of ivermectin (LT_50_ = 20.89 h) (Table 2). The above results showed that **1** possessed a much faster acaricidal action than ivermectin.

**Scheme 2:**
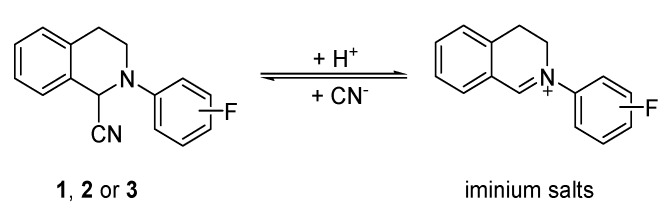
Transformation between **1**, **2** or **3** and its corresponding iminium form.

In our previous study, 6-alkoxydihydrosanguinarines were proven to be prodrugs of sanguinarine for the acaricidal activity [9]. 6-Alkoxydihydrosanguinarines, which structurally contains a *N*,*O*-acetal moiety, are very easily converted to the corresponding iminium ion, (*i.e.*, sanguinarine) under acidic conditions [18] including the acidic medium of the lysosome of cells [19,20]. Like the 6-alkoxy-dihydrosanguinarines, 1-cyano-2-aryl-1,2,3,4-tetrahydroisoquinolines (CATHIQs) also have hydrolyzable properties under acidic condition (pH < 6.0). We observed that dissolving compounds **1**, **2**, or **3** in a buffer solution (pH = 4.3) at room temperature for 25 min resulted in an equilibrium between the compound and its corresponding iminium form (Scheme 2).

At that time, the molar ratio of **1** to its corresponding iminium form was about 3:2 (unpublished data). In view of the structural similarity of CATHIQs, we speculated that similar hydrolysable properties should exist in the other CATHIQs. Considering the acidic conditions (pH = 3.5–5.5) in the lysosome of cells, the above transformation of CATHIQs perhaps also occurs in cells. The above analysis suggests that CATHIQs may be prodrugs or precursor drugs of their corresponding iminium salts (ADHIQs). This will be further investigated in detail in our future work. The activity of each CATHIQ listed in Figure 3 is lower than that of its corresponding iminium salt (ADHIQ) [10], which may be related with that various CATHIQs have different hydrolysable properties under acidic conditions, such as hydrolysis rates and equilibrium constants. In fact, we indeed observed that compounds **1**, **2** and **3** had different susceptibility to the same acidic solutions in the range of pH 2.2–6.3.

As potential drugs, CATHIQs have higher compatibility with physiological conditions than the corresponding ADHIQs because we have proven that they have an ability to resist some biological reducing agents such as NAD(P)H and nucleophiles with amino or mercapto groups. Based on the considerations above, the present research strongly suggests that the target compounds represent a class of novel promising candidates or lead compounds for the development of new tetrahydroisoquinoline acaricidal agents. At present, *in vivo* testing of the CATHIQs is underway in our lab, and the corresponding results will be reported later.

## 3. Experimental Section

### 3.1. Materials and Apparatus

Ivermectin (C_48_H_74_O_14_, molecular weight: 875.09276, ≥91%, 22,23-dihydroavermectin B1 consisting of 95% avermectin B_1a_ and 5% avermectin B_1b_) was purchased from Sigma-Aldrich Trading Co. Ltd. (Shanghai, China). Other chemicals used in the present study were purchased from J&K Chemical Ltd. (Beijing, China) and used without further purification. The intermediate 2-aryl-3,4-dihydroisoquinolin-2-ium bromides (ADHIQs in Figure 1) were prepared according to our previously reported method [10]. Melting points (mp) were determined on an XT-4 micro-melting point apparatus and are uncorrected. ^1^H-NMR and ^13^C-NMR spectra were recorded with a Bruker AVANCE III instrument operating at 500 and 125 MHz, respectively, and using TMS as an internal standard. ESI-MS were measured on a Trace mass spectrometer.

### 3.2. General Procedure for the Synthesis of Compounds ***1**–**24***

To the solution of 2-aryl-3,4-dihydroisoquinolin-2-ium bromine (0.5 mmol) in ethanol or 95% ethanol in water (ca. 20 mL), KCN (76 mg, 1.17 mmol) was added. The reaction solution was stirred at room temperature or 0 °C for 30 min, and then diluted with CHCl_3_ or CH_2_Cl_2_ (80 mL). The resulting solution was washed with water (3 × 30 mL), and dried over anhydrous sodium sulfate. The solvent was removed under a reduced pressure to provide crude product as an oil or solid. The crude product was subjected to column chromatography over silica gel eluting with the mixture of petroleum ether and ethyl acetate (10:1) to afford **1**–**24** as solids or liquids.

*2-(2-Fluorophenyl)-1,2,3,4-tetrahydroisoquinoline-1-carbonitrile* (**1**): white solid; yield: 74%; m.p. 88–89 °C; IR (KBr) *υ*_max_ cm^−1^: 2224 (w, C≡N), 1142 (s, C−N), 1228 (s, C−F); UV (MeOH): λ_max_ (lg *ε*) 237(4.09) nm; ^1^H-NMR (CDCl_3_) *δ*: 7.29–7.33 (1H, m), 7.25–7.27 (2H, m), 7.21–7.23 (2H, m), 7.16–7.19 (1H, m), 7.10–7.12 (2H, m), 5.48 (1H, s, H-1), 3.60–3.51 (2H, m, H-3), 3.22 (1H, ddd, *J* = 17.0, 11.0, 7.0 Hz, H-4a), 2.93 (1H, d-like, H-4b); ^13^C-NMR (CDCl_3_) *δ*: 156.1 (d, *J* = 245.0 Hz, C-2'), 136.9 (d, *J* = 9.2 Hz, C-1'), 134.0, 129.6, 129.3, 128.7, 127.1, 126.8, 125.0 (d, *J* = 4.4 Hz), 121.5 (d, *J* = 2.0 Hz), 117.5 (C≡N), 116.4 (d, *J* = 20.2 Hz), 53.9 (C-1), 44.8 (C-3), 28.6 (C-4); positive ESI-MS *m/z*: 226 [M−CN]^+^, 253 [M+H]^+^, 275 [M+Na]^+^. The IR, ^1^H-NMR and ^13^C-NMR data are agreement with the literature data [17].

Crystallographic data and X-ray structure analysis of **1**. A colorless crystal (0.50 × 0.28 × 0.24) of **1** was grown by slow evaporation in petroleum ether-AcOEt solution. Diffraction intensity data were acquired with a CCD diffractometer with graphite-monochromated Mo*Ká* radiation (λ = 0.71073 Å). Crystal data for **1**: C_16_H_13_FN_2_, *M*_r_ = 252.28, Orthorhombic, space group Pbca, Z = 8, *a* = 7.4686(7), *b* = 14.9034(14), *c* = 23.239(2) Å, *α* = 90.00°, *β* = 90.00°, *γ* = 90.00°, *V* = 2586.7(4) Å^3^, *T* = 296(2) K, *μ* (Mo Ká) = 0.087 mm^−1^, 17836 reflections measured, 2402 independent reflections (*R*_int_ = 0.0252), *S* = 0.993. The final *R*_1_ values were 0.0363 and *wR*_2_ = 0.1182 (*I* > 2*σ* (*I*)). The final *R*_1_ values were 0.0472 and *wR*_2_ = 0.1324 (for all data). Crystallographic data for **1** have been deposited with the Cambridge Crystallographic Data Centre (deposition number CCDC 986701). Copies of the data can be obtained, free of charge, on application to the Director, CCDC, 12 Union Road, Cambridge CB21EZ, UK (fax: +44(0)-1223–336033 or e-mail: deposit@ccdc.cam. ac.uk).

*2-(3-Fluorophenyl)-1,2,3,4-tetrahydroisoquinoline-1-carbonitrile* (**2**): white solid; yield: 83%; m.p. 94–95 °C; IR (KBr) *υ*_max_ cm^−1^: 2219 (w, C≡N, 1146 (s, C−N), 1174 (s, C−F); UV (MeOH): λ_max_ (lg *ε*) 246 (4.12) nm; ^1^H-NMR (CDCl_3_) *δ*: 7.28–7.37 (5H, m), 6.85 (1H, dd, *J* = 8.5, 2.5 Hz), 6.79 (1H, dt, *J* = 11.5, 2.5 Hz), 6.73 (1H, td, *J* = 8.5, 2.5 Hz), 5.54 (1H, s, H-1), 3.78–3.83 (1H, m, H-3a), 3.53 (1H, ddd, *J* = 12.5, 10.5, 4.0 Hz, H-3b), 3.19 (1H, ddd, *J* = 16.5, 10.5, 6.0 Hz, H-4a), 3.05 (1H, dt, *J* = 16.0, 4.0 Hz, H-4b); ^13^C-NMR (CDCl_3_) *δ*: 163.8 (d, *J* = 243.6 Hz, C-3'), 149.8 (d, *J* = 9.6 Hz, C-1'), 134.6, 130.8 (d, *J* = 9.9 Hz), 129.3, 129.0, 127.1, 117.5 (C≡N), 112.2 (d, *J* = 2.6 Hz), 108.1 (d, *J* = 21.1 Hz), 104.1 (d, *J* = 25.1 Hz), 52.3 (C-1), 44.1 (C-3), 28.4 (C-4); positive ESI-MS *m/z*: 226 [M−CN]^+^, 253 [M+H]^+^, 275 [M+Na]^+^. HR-MS [M+Na]^+^: Calcd. for C_16_H_13_FN_2_Na^+^, 275.0960, found 275.0969.

*2-(4-Fluorophenyl)-1,2,3,4-tetrahydroisoquinoline-1-carbonitrile* (**3**): white solid; yield: 88%; m.p. 124–125 °C; IR (KBr) *υ*_max_ cm^−1^: 2226 (w, C≡N), 1142 (s, C−N), 1244 (s, C−F); UV (MeOH): λ_max_ (lg *ε*) 240 (3.97) nm; ^1^H-NMR (CDCl_3_) *δ*: 7.23–7.34 (4H, m), 7.08 (2H, s), 7.07 (2H, d, *J* = 2.5 Hz), 5.40 (1H, s, H-1), 3.63 (1H, q-like, H-3a), 3.46 (1H, td, *J* = 11.0, 4.0 Hz, H-3b), 3.17 (1H, ddd, *J* = 17.0, 11.0, 6.5 Hz, H-4a), 2.96 (1H, dt, *J* = 16.0, 3.5 Hz, H-4b); ^13^C-NMR (CDCl_3_) *δ*: 158.6 (d, *J* = 240.4 Hz, C-4'), 145.1 (d, *J* = 2.5 Hz, C-1'), 134.3, 129.5, 129.4, 128.8, 127.1, 126.9, 120.5 (d, *J* = 10.5 Hz), 117.4 (C≡N), 116.2 (d, *J* = 22.4 Hz), 54.8 (C-1), 44.8 (C-3), 28.6 (C-4); positive ESI-MS *m/z*: 226 [M−CN]^+^, 253 [M+H]^+^, 275 [M+Na]^+^. The IR, ^1^H-NMR and ^13^C-NMR data are agreement with the literature data [17].

*2-(2-Chlorophenyl)-1,2,3,4-tetrahydroisoquinoline-1-carbonitrile* (**4**): white sheet-like crystals; yield: 87%; m.p. 115–116 °C; IR (KBr) *υ*_max_ cm^−1^: 2224 (w, C≡N), 1142 (s, C−N); UV (MeOH): λ_max_ (lg *ε*) 243 (3.92) nm; ^1^H-NMR (CDCl_3_) *δ*: 7.44 (1H, d-like, *J* = 8.0 Hz), 7.30–7.35 (3H, m), 7.23–7.27 (3H, m), 7.13–7.16 (1H, m), 5.53 (1H, s, H-1), 3.62 (1H, td, *J* = 12.0, 4.0 Hz, H-3a), 3.46 (1H, dd, *J* = 12.0, 6.5 Hz, H-3b), 3.26 (1H, ddd, *J* = 16.5, 11.5, 6.0 Hz, H-4a), 2.93 (1H, dd, *J* = 16.5, 2.5 Hz, H-4b); ^13^C-NMR (CDCl_3_) *δ*: 145.9 (C-1'), 134.1, 130.7, 129.7, 128.7, 128.2, 127.1, 126.7, 126.1, 123.2, 117.4 (C≡N), 53.9 (C-1), 45.6 (C-3), 28.8 (C-4); positive ESI-MS *m/z*: 242 [M−CN]^+^, 269 [M+H]^+^, 291[M+Na]^+^. The IR, ^1^H-NMR and ^13^C-NMR are in agreement with the literature data [17].

*2-(3-Chlorophenyl)-1,2,3,4-tetrahydroisoquinoline-1-carbonitrile* (**5**): white powder; yield: 78%; m.p. 83–85 °C; IR (KBr) *υ*_max_ cm^−1^: 2223 (w, C≡N), 1153 (s, C−N); UV (MeOH): λ_max_ (lg *ε*) 251 (4.41) nm; ^1^H-NMR (CDCl_3_) *δ*: 7.23–7.32 (5H, m), 7.03 (1H, t-like), 6.92–6.97 (2H, m), 5.49 (1H, s, H-1), 3.72–3.76 (1H, m, H-3a), 3.48 (1H, ddd, *J* = 12.5, 10.5, 4.5 Hz, H-3b), 3.12 (1H, ddd, *J* = 16.0, 10.0, 5.5 Hz, H-4a), 2.97 (1H, dt, *J* = 16.5, 4.0 Hz, H-4b); ^13^C-NMR (CDCl_3_) *δ*: 149.4 (C-1'), 135.4, 134.5, 130.6, 129.3, 129.2, 129.0, 127.1, 121.5, 117.5 (C≡N), 117.2, 115.0, 52.4 (C-1), 44.1 (C-3), 28.4 (C-4); positive ESI-MS *m/z*: 242[M−CN]^+^, 269.0[M+H]^+^, 291[M+Na]^+^. The IR, ^1^H-NMR and ^13^C-NMR data are agreement with the literature data [17].

*2-(4-Chlorophenyl)-1,2,3,4-tetrahydroisoquinoline-1-carbonitrile* (**6**): white powder; yield: 78%; m.p. 152–153 °C; IR (KBr) *υ*_max_ cm^−1^: 2223 (w, C≡N), 1142 (s, C−N); UV (MeOH): λ_max_ (lg *ε*) 253 (4.59) nm; ^1^H-NMR (CDCl_3_) *δ*: 7.31–7.35 (5H, m), 7.27–7.28 (1H, m), 7.02–7.05 (2H, m), 5.49 (1H, s, H-1), 3.74 (1H, ddd-like, H-3a), 3.50 (1H, ddd-like, H-3b), 3.18 (1H, ddd, *J* = 16.0, 10.5, 6.0 H-4a), 3.00 (1H, dt, *J* = 16.0, 3.5 Hz, H-4b); ^13^C-NMR (CDCl_3_) *δ*: 147.0 (C-1'), 134.4, 129.6, 129.4, 129.3, 128.9, 127.1, 127.0, 118.9, 117.5 (C≡N), 53.1 (C-1), 44.3 (C-3), 28.5 (C-4); positive ESI-MS *m/z*: 242 [M−CN]^+^. The IR, ^1^H-NMR and ^13^C-NMR data are agreement with the literature data [17].

*2-(2-Bromophenyl)-1,2,3,4-tetrahydroisoquinoline-1-carbonitrile* (**7**): white powder; yield: 90%; m.p. 119–120 °C; IR (KBr) *υ*_max_ cm^−1^: 2224 (w, C≡N); 1141 (s, C−N); UV (MeOH): λ_max_ (lg *ε*) 240 (4.12) nm; ^1^H-NMR (CDCl_3_) *δ*: 7.62 (1H, dd, *J* = 7.5, 1.0 Hz), 7.40 (1H, td-like), 7.30–7.35 (2H, m), 7.23–7.27 (3H, m), 5.53 (1H, s, H-1), 3.64 (1H, dt, *J* = 12.0, 4.0 Hz, H-3a), 3.42 (1H, dd, *J* = 12.0, 6.5 Hz, H-3b), 3.27 (1H, ddd, *J* = 17.0, 11.5, 6.5 Hz, H-4a), 2.92 (1H, dd, *J* = 16.5, 3.0 Hz, H-4b); ^13^C-NMR (CDCl_3_) *δ*: 147.2 (C-1'), 134.2, 133.8, 129.7, 129.5, 128.9, 128.7, 127.1, 126.7, 123.8, 120.5, 117.3(C≡N), 54.3 (C-1), 45.8 (C-3), 28.9 (C-4); positive ESI-MS *m/z*: 286 [M−CN]^+^, 313 [M+H]^+^. HR-MS [M+Na]^+^: Calcd. for C_16_H_13_BrN_2_Na^+^, 335.0160, found 335.0155.

*2-(3-Bromophenyl)-1,2,3,4-tetrahydroisoquinoline-1-carbonitrile* (**8**): white sheet crystal; yield: 75%; m.p. 92–93 °C; IR (KBr) *υ*_max_ cm^−1^: 2220 (w, C≡N), 1150 (s, C−N); UV (MeOH): λ_max_ (lg *ε*) 252 (4.13) nm; ^1^H-NMR (CDCl_3_) *δ*: 7.31–7.37 (3H, m), 7.24–7.29 (3H, m), 7.15–7.17 (1H, m), 7.02–7.03 (1H, m), 5.53 (1H, s, H-1), 3.77–3.81 (1H, m, H-3a), 3.53 (1H, td, *J* = 11.5, 4.0 Hz, H-3b), 3.18 (1H, ddd, *J* = 16.0, 10.5, 6.0 Hz, H-4a), 3.02 (1H, d, *J* = 16.5 Hz, H-4b); ^13^C-NMR (CDCl_3_) *δ*: 149.5 (C-1'), 134.5, 130.9, 129.4, 129.2, 129.0, 127.1, 127.0, 124.4, 123.6, 120.1, 117.5 (C≡N), 115.4, 52.4 (C-1), 44.1 (C-3), 28.4 (C-4); positive ESI-MS *m/z*: 286[M−CN]^+^. HR-MS [M+Na]^+^: Calcd. for C_16_H_13_BrN_2_Na^+^, 335.0160, found 335.0154.

*2-(4-Bromophenyl)-1,2,3,4-tetrahydroisoquinoline-1-carbonitrile* (**9**): white sheet crystal; yield: 72%; m.p. 156–157 °C; IR (KBr) *υ*_max_ cm^−1^: 2222 (w, C≡N), 1138 (s, C−N); UV (MeOH): λ_max_ (lg *ε*) 204 (4.70), 255 (4.48) nm; ^1^H-NMR (CDCl_3_) *δ*: 7.45 (2H, d-like, *J* = 8.5 Hz), 7.28–7.34 (3H, m), 7.23–7.25 (1H, m), 6.95 (2H, d-like, *J* = 9.0 Hz), 5.45 (1H, s, H-1), 3.69–3.74 (1H, m, H-3a), 3.46 (1H, ddd, *J* = 12.5, 10.5, 4.0 Hz, H-3b), 3.17 (1H, ddd, *J* = 16.5, 10.5, 6.0 Hz, H-4a), 2.98 (1H, dt, *J* = 13.0, 3.5 Hz, H-4b); ^13^C-NMR (CDCl_3_) *δ*: 147.4 (C-1'), 134.4, 132.5, 129.4, 129.2, 129.0, 127.1, 127.0, 119.1, 117.5 (C≡N), 114.4, 52.9 (C-1), 44.3 (C-3), 28.4 (C-4); positive ESI-MS *m/z*: 286 [M−CN]^+^, 313 [M+H]^+^. The IR, ^1^H-NMR and ^13^C-NMR data are agreement with the literature data [17].

*2-(2-Iodophenyl)-1,2,3,4-tetrahydroisoquinoline-1-carbonitrile* (**10**): white powder; yield: 83%; m.p. 127–128 °C; IR (KBr) *υ*_max_ cm^−1^: 2223 (w, C≡N), 1138 (s, C−N); UV (MeOH): λ_max_ (lg *ε*) 204 (4.01) nm; ^1^H-NMR (CDCl_3_) *δ*: 7.90 (1H, d, *J* = 7.5 Hz), 7.42–7.45 (1H, t-like, *J* = 7.5 Hz), 7.31–7.34 (2H, m), 7.23–7.27 (3H, m), 6.95 (1H, t, *J* = 7.5 Hz, H-6'), 5.43 (1H, s, H-1), 3.63–3.68 (1H, t-like, *J* = 11.5 Hz, H-3a), 3.35–3.38 (1H, m, H-3b), 3.23–3.30 (1H, m, H-4a), 2.92 (1H, d, *J* = 16.5 Hz, H-4b); ^13^C-NMR (CDCl_3_) *δ*: 150.0 (C-1'), 140.1, 134.3, 129.8, 129.7, 129.5, 128.7, 127.6, 127.1, 126.7, 123.9, 117.3 (C≡N), 98.5 (C-2'), 55.1 (C-1), 46.2 (C-3), 29.1 (C-4); positive ESI-MS *m/z*: 334 [M−CN]^+^, 361 [M+H]^+^, 383 [M+Na]^+^. HR-MS [M+Na]^+^: Calcd. for C_16_H_13_IN_2_Na^+^, 383.0021, found 383.0015.

*2-(3-Iodophenyl)-1,2,3,4-tetrahydroisoquinoline-1-carbonitrile* (**11**): white powder; yield: 79%; m.p. 124–125 °C; IR (KBr) *υ*_max_ cm^−1^: 2220 (w, C≡N), 1135 (s, C−N); UV (MeOH): λ_max_ (lg *ε*) 252 (4.30) nm; ^1^H-NMR (CDCl_3_) *δ*: 7.39 (1H, s, H-2’), 7.29–7.33 (4H, m), 7.24–7.25 (1H, m), 7.01–7.08 (2H, m), 5.47 (1H, s, H-1), 3.72–3.76 (1H, m, H-3a), 3.45–3.51 (1H, m, H-3b), 3.14 (1H, ddd, *J* = 16.5, 10.5, 6.0 Hz, H-4a), 2.95 (1H, dt, *J* = 16.0, 4.0 Hz, H-4b); ^13^C-NMR (CDCl_3_) *δ*: 149.5 (C-1'), 134.5, 131.0, 130.6, 129.3, 129.2, 129.0, 127.1, 126.2, 117.5 (C≡N), 116.2, 95.3, 52.5 (C-1), 44.1 (C-3), 28.4 (C-4); positive ESI-MS *m/z*: 334 [M−CN]^+^, 383 [M+Na]^+^. HR-MS [M+Na]^+^: Calcd. for C_16_H_13_IN_2_Na^+^, 383.0021, found 383.0017.

*2-(4-Iodophenyl)-1,2,3,4-tetrahydroisoquinoline-1-carbonitrile* (**12**): white powder; yield: 88%; m.p. 145–147 °C; IR (KBr) *υ*_max_ cm^−1^: 2221 (w, C≡N), 1140 (s, C−N); UV (MeOH): λ_max _(lg *ε*) 258 (4.39) nm; ^1^H-NMR (CDCl_3_) *δ*: 7.66 (2H, d-like, *J* = 9.0 Hz, H-3', H-5'), 7.31–7.34 (3H, m), 7.26–7.28 (1H, m), 6.86 (2H, d-like, *J* = 9.0 Hz, H-2', H-6'), 5.49 (1H, s, H-1), 3.73–3.78 (1H, m, H-3a), 3.47–3.52 (1H, m, H-3b), 3.17 (1H, ddd, *J* = 16.5, 10.5, 6.0 Hz, H-4a), 3.01 (1H, dt, *J* = 16.0, 4.0 Hz, H-4b); ^13^C-NMR (CDCl_3_) *δ*: 148.0, 138.4 (C-3', C-5'), 134.5, 129.4, 129.2, 129.0, 127.1, 127.0, 119.3, 117.5 (C≡N), 84.2, 52.5 (C-1), 44.1 (C-3), 28.4 (C-4); positive ESI-MS *m/z*: 334 [M−CN]^+^, 361 [M+H]^+^, 383 [M+Na]^+^. HR-MS [M+Na]^+^: Calcd. for C_16_H_13_IN_2_Na^+^, 383.0021, found 383.0014.

*2-(2-(Trifluoromethyl)phenyl)-1,2,3,4-tetrahydroisoquinoline-1-carbonitrile* (**13**): white powder; yield: 90%; m.p. 101–102 °C; IR (KBr) *υ*_max_ cm^−1^: 2224 (w, C≡N), 1169 (s, C−N), 1312 (s, C−F); UV (MeOH): λ_max_ (lg *ε*) 245 (4.05) nm; ^1^H-NMR (CDCl_3_) *δ*: 7.70 (2H, d, *J* = 8.0 Hz), 7.65 (1H, t, *J* = 7.5 Hz), 7.39 (1H, t, *J* = 7.5 Hz), 7.31 (1H, t, *J* = 7.5 Hz), 7.20–7.26 (3H, m), 5.13 (1H, s, H-1), 3.65–3.71 (1H, m, H-3a), 3.20–3.28 (2H, m, H-3b, H-4a), 2.87–2.91 (1H, m, H-4b); ^13^C-NMR (CDCl_3_) *δ*: 148.3, 134.1, 133.4, 129.8, 129.7, 128.6, 127.3 (q, *J* = 5.0 Hz), 126.9 (d, *J* = 7.5 Hz), 126.6, 126.4, 117.9 (C≡N), 56.2 (C-1), 47.2 (C-3), 28.9 (C-4); positive ESI-MS *m/z*: 276 [M−CN]^+^, 325 [M+Na]^+^. HR-MS [M+Na]^+^: Calcd. for C_17_H_13_F_3_N_2_Na^+^, 325.0929, found 325.0933.

*2-(3-(Trifluoromethyl)phenyl)-1,2-dihydroisoquinoline-1-carbonitrile* (**14**): white crystal; yield: 93%; m.p. 100–101 °C; IR (KBr) *υ*_max_ cm^−1^: 2226 (w, C≡N), 1166 (s, C−N), 1327 (s, C−F); UV (MeOH): λ_max_ (lg *ε*) 251 (4.64) nm; ^1^H-NMR (CDCl_3_) *δ*: 7.47 (1H, t, *J* = 8.0 Hz), 7.30–7.35 (3H, m), 7.22–7.27 (4H, m), 5.54 (1H, s, H-1), 3.79–3.83 (1H, m, H-3a), 3.54 (1H, td-like, *J* = 10.0, 4.0 Hz, H-3b), 3.17 (1H, ddd, *J* = 16.0, 10.0, 6.0 Hz, H-4a), 3.02 (1H, dt, *J* = 16.5, 4.0 Hz, H-4b); ^13^C-NMR (CDCl_3_) *δ*: 148.5, 134.4, 132.0 (d, *J* = 32.0 Hz), 130.2, 129.4, 129.1, 129.0, 119.8, 118.0, 117.4 (C≡N), 113.7, 52.4 (C-1), 44.2 (C-3), 28.4 (C-4); positive ESI-MS *m/z*: 276 [M−CN]^+^. HR-MS [M+Na]^+^: Calcd. for C_17_H_13_F_3_N_2_Na^+^, 325.0929, found 325.0939.

*2-(4-(Trifluoromethyl)phenyl)-1,2,3,4-tetrahydroisoquinoline-1-carbonitrile* (**15**): white powder; yield: 98%; m.p. 101–102 °C; IR (KBr) *υ*_max_ cm^−1^: 2224 (w, C≡N), 1172 (s,C−N), 1326 (s, C−F); UV (MeOH): λ_max _(lg *ε*) 205 (4.40), 259 (4.32) nm; ^1^H-NMR (CDCl_3_) *δ*: 7.61 (2H, d, *J* = 8.5 Hz, H-3', H-5'), 7.26–7.37 (4H, m), 7.09 (2H, d, *J* = 8.5 Hz, H-2', H-6'), 5.58 (1H, s, H-1), 3.84–3.88 (1H, m, H-3a), 3.55–3.60 (1H, m, H-3b), 3.17 (1H, ddd, *J* = 16.0, 10.0, 6.0 Hz, H-4a), 3.06 (1H, dt, *J* = 16.0, 8.5, 4.5 Hz, H-4b); ^13^C-NMR (CDCl_3_) *δ*: 150.4 (C-1'), 134.6, 129.3, 129.1, 129.1, 127.2, 127.1, 126.9, 126.9, 117.5 (C≡N), 115.4, 51.3 (C-1), 44.0 (C-3), 28.3 (C-4); positive ESI-MS *m/z*: 276 [M−CN]^+^, 325 [M+Na]^+^. HR-MS [M+Na]^+^: Calcd. for C_17_H_13_F_3_N_2_Na^+^, 325.0929, found 325.0935.

*2-(3-Nitrophenyl)-1,2,3,4-tetrahydroisoquinoline-1-carbonitrile* (**16**): yellow powder; yield: 90%; m.p. 168–170 °C; IR (KBr) *υ*_max_ cm^−1^: 2224 (w, C≡N), 1149 (s, C−N), 1579 (s, NO_2_); UV (MeOH): λ_max_ (lg *ε*) 211 (4.13), 247 (4.31) nm; ^1^H-NMR (CDCl_3_) *δ*: 7.89 (1H, s, H-2'), 7.83 (1H, d, *J* = 8.0 Hz), 7.52 (1H, t, *J* = 8.0 Hz), 7.32–7.36 (4H, m), 7.26–7.29 (1H, m), 5.60 (1H, s, H-1), 3.85–3.89 (1H, m, H-3a), 3.57–3.62 (1H, m, H-3b), 3.19 (1H, ddd, *J* = 16.0, 10.0, 6.0 Hz, H-4a), 3.08 (1H, dt, *J* = 16.0, 4.0 Hz, H-4b); ^13^C-NMR (CDCl_3_) *δ*: 148.9 (C-1'), 134.4 (C-3'), 130.4, 129.3, 129.2, 128.8, 127.3, 127.1, 121.7, 117.2 (C≡N), 115.7, 111.0, 51.7 (C-1), 44.1 (C-3), 28.3 (C-4); positive ESI-MS *m/z*: 253 [M−CN]^+^, 302 [M+Na]^+^. HR-MS [M+Na]^+^: Calcd. for C_16_H_13_N_3_NaO_2_^+^, 302.0905, found 302.0900.

*2-(4-Nitrophenyl)-1,2,3,4-tetrahydroisoquinoline-1-carbonitrile* (**17**): yellow powder; yield: 93%; m.p. 102–104 °C; IR (KBr) *υ*_max_ cm^−1^: 2361 (w, C≡N), 1162 (s, C−N), 1597 (s, NO_2_); UV (MeOH): λ_max_ (lg *ε*) 368 (4.00) nm; ^1^H-NMR (CDCl_3_) *δ*: 8.16–8.18 (2H, d, *J* = 9.0 Hz, H-2', H-6'), 7.25–7.33 (4H, m), 6.94–6.98 (2H, d, *J* = 8.5 Hz, H-3', H-5'), 5.79 (1H, s, H-1), 3.77–3.81 (1H, m, H-3a), 3.50–3.56 (1H, m, H-3b), 3.38–3.44 (1H, m, H-4a), 2.97 (1H, dt, *J* = 15.0, 5.0 Hz, H-4b); ^13^C-NMR (CDCl_3_) *δ*: 153.6 (C-1'), 138.9 (C-4'), 135.7, 133.6, 129.0, 128.0, 126.5, 126.1, 126.0, 113.4 (C≡N), 112.3, 61.4 (C-1), 43.7 (C-3), 27.8 (C-4); positive ESI-MS *m/z*: 253 [M−CN]^+^. HR-MS [M+Na]^+^: Calcd. for C_16_H_13_N_3_NaO_2_^+^, 302.0905, found 302.0901.

*2-(2-Methylphenyl)-1,2,3,4-tetrahydroisoquinoline-1-carbonitrile* (**18**): yellow crystal; yield: 82%; m.p. 136–138 °C; IR (KBr) *υ*_max_ cm^−1^: 2220 (w, C≡N), 1141 (s, C−N); UV (MeOH): λ_max_ (lg *ε*) 237 (3.88) nm; ^1^H-NMR (CDCl_3_) *δ*: 7.22–7.31 (7H, m), 7.14–7.11 (1H, m), 5.06 (1H, s, H-1), 3.61 (1H, td, *J* = 11.4, 3.8 Hz, H-3a), 3.35–3.32 (1H, m, H-3b), 3.18 (1H, ddd, *J* = 16.8, 11.4, 6.2 Hz, H-4a), 2.92 (1H, d-like, *J* = 16.8 Hz, H-4b), 2.29 (3H, s); ^13^C-NMR (CDCl_3_) * δ*: 148.0 (C-1'), 134.5, 133.4, 131.2, 130.1, 129.7, 128.6, 127.2, 127.0, 126.6, 125.4, 122.0, 117.7 (C≡N), 55.6 (C-1), 44.9 (C-3), 28.7 (C-4); positive ESI-MS *m/z*: 222 [M−CN]^+^. HR-MS [M+Na]^+^: Calcd. for C_17_H_16_N_2_Na^+^, 271.1211, found 271.1207.

*2-(3-Methylphenyl)-1,2,3,4-tetrahydroisoquinoline-1-carbonitrile* (**19**): yellow oil; yield: 75%; IR (KBr) *υ*_max_ cm^−1^: 2224 (w, C≡N), 1030 (vs, C−N); UV (MeOH): λ_max _(lg *ε*) 247 (4.12) nm; ^1^H-NMR (CDCl_3_) *δ*: 7.28–7.20 (5H, m), 6.88–6.86 (2H, m), 6.82 (1H, d, *J* = 7.5 Hz, H-4'), 5.49 (1H, s, H-1), 3.74 (1H, ddd, *J* = 12.4, 6.0, 2.9 Hz, H-3a), 3.44 (1H, ddd, *J* = 12.4, 10.9, 4.1 Hz, H-3b), 3.12 (1H, ddd, *J* = 16.4, 10.9, 6.0 Hz, H-4a), 2.92 (1H, dt, *J* = 16.4, 3.4 Hz, H-4b), 2.35 (3H, s); ^13^C-NMR (CDCl_3_) *δ*: 148.5 (C-1'), 139.4, 134.7, 129.7, 129.5, 129.4, 128.8, 127.1, 126.9, 122.8, 118.4, 117.9 (C≡N), 114.7, 53.3 (C-1), 44.2 (C-3), 28.6 (C-4); positive ESI-MS *m/z*: 222[M−CN]^+^. The IR, ^1^H-NMR and ^13^C-NMR data are agreement with the literatural data [17].

*2-(4-Methylphenyl)-1,2,3,4-tetrahydroisoquinoline-1-carbonitrile* (**20**): yellow solid; yield: 64%; m.p. 114–116 °C; IR (KBr) *υ*_max_ cm^−1^: 2222 (w, C≡N), 1137 (s, C−N); UV (MeOH): λ_max_ (lg *ε*) 245 (4.11) nm; ^1^H-NMR (CDCl_3_) * δ*: 7.30–7.21 (4H, m), 7.16 (2H, d, *J* = 8.2 Hz, H-3', 5'), 7.00 (2H, d, *J* = 8.4 Hz, H-2', 6'), 5.45 (1H, s, H-1), 3.70 (1H, ddd, *J* = 12.4, 6.1, 2.3 Hz, H-3a), 3.44 (1H, ddd, *J* = 12.4, 11.2, 4.0 Hz, H-3b), 3.15 (1H, ddd, *J* = 16.7, 11.2, 6.1 Hz, H-4a), 2.94 (1H, dt, *J* = 16.7, 3.2 Hz, H-4b), 2.31 (3H, s); ^13^C-NMR (CDCl_3_) *δ*: 146.3 (C-1'), 134.6, 131.8, 130.1 (C-3', 5'), 129.7, 129.4, 128.7, 127.1, 126.8, 118.3 (C-2', 6'), 117.7 (C≡N), 114.8, 54.1 (C-1), 44.4 (C-3), 28.6 (C-4); positive ESI-MS *m/z*: 222 [M−CN]^+^. The IR, ^1^H-NMR and ^13^C-NMR data are agreement with the literature data [17].

*2-(2-Methoxyphenyl)-1,2,3,4-tetrahydroisoquinoline-1-carbonitrile* (**21**): white solid; yield: 67%; m.p. 150–152°C; IR (KBr) *υ*_max_ cm^−1^: 2223 (w, C≡N), 1144 (s, C−N), 1243 (vs, C-O-C); UV (MeOH): λ_max_ (lg *ε*) 241 (3.84), 278 (3.48) nm; ^1^H-NMR (CDCl_3_) *δ*: 7.12–7.30 (6H, m), 7.02 (1H, td, *J* = 8.0, 1.0 Hz,), 6.92 (1H, d, *J* = 8.0 Hz), 5.74 (1H, s, H-1), 3.85 (3H, s), 3.52 (2H, dd, *J* = 8.0 Hz, H-3), 3.20–3.26 (1H, m, H-4a), 2.92 (1H, dt, *J* = 16.5, 2.5 Hz, H-4b); ^13^C-NMR (CDCl_3_) *δ*: 152.5 (C-2'), 137.8 (C-1'), 134.1, 129.9, 129.6, 128.5, 127.2, 126.6, 125.1, 121.5, 121.0, 117.8 (C≡N), 111.5, 55.6 (C-1), 53.2, 44.8 (C-3), 28.8 (C-4); positive ESI-MS *m/z*: 238 [M−CN]^+^, 265 [M+H]^+^, 287 [M+Na]^+^. The IR, ^1^H-NMR and ^13^C-NMR data are agreement with the literature data [17].

*2-(3-Methoxyphenyl)-1,2,3,4-tetrahydroisoquinoline-1-carbonitrile* (**22**): yellow oil; yield: 73%; IR (KBr) *υ*_max_ cm^−1^: 2221(w, C≡N), 1165 (s, C−N), 1212 (vs, C-O-C)); UV (MeOH): λ_max_ (lg *ε*) 245 (4.09) nm; ^1^H-NMR (CDCl_3_) *δ*: 7.21–7.31 (5H, m), 6.54–6.68 (3H, m), 5.51 (1H, s, H-1), 3.81 (3H, s), 3.74–3.77 (1H, m, H-3a), 3.43–3.49 (1H, m, H-3b), 3.10–3.16 (1H, m, H-4a), 2.95 (1H, d, *J* = 16.5 Hz, H-4b); ^13^C-NMR (CDCl_3_) *δ*: 160.8 (C-3'), 149.7 (C-1'), 134.7, 130.4, 129.6, 129.4, 128.8, 127.1, 126.9, 117.8 (C≡N), 109.9, 106.6, 104.0, 55.4(C-1), 53.0, 44.2 (C-3), 28.5 (C-4); positive ESI-MS *m/z*: 238 [M−CN]^+^, 265 [M+H]^+^. The IR, ^1^H-NMR and ^13^C-NMR data are agreement with the literature data [17].

*2-(4-Methoxyphenyl)-1,2,3,4-tetrahydroisoquinoline-1-carbonitrile* (**23**): yellow oil; yield: 78%; IR (KBr) *υ*_max_ cm^−1^: 2222 (w, C≡N), 1136 (s, C−N), 1248 (vs, C-O-C); UV (MeOH): λ_max_ (lg *ε*) 242 (4.05) nm; ^1^H-NMR (CDCl_3_) *δ*: 7.21–7.30 (4H, m), 7.08 (2H, d, *J* = 9.0 Hz, H-3', H-5'), 6.91 (2H, d, *J* = 9.0 Hz, H-2', H-6'), 5.36 (1H, s, H-1), 3.79 (3H, s), 3.55–3.59 (1H, m, H-3a), 3.42 (1H, td, *J* = 11.5, 3.5 Hz, H-3b), 3.15 (1H, ddd, *J* = 16.5, 11.5, 6.5 Hz, H-4a), 2.92 (1H, d, *J* = 16.0 Hz, H-4b); ^13^C-NMR (CDCl_3_) *δ*: 155.7 (C-4'), 142.6 (C-1'), 134.4, 129.7, 129.5, 129.0, 127.1, 126.7, 121.0, 117.7 (C≡N), 114.8, 55.6 (C-1), 44.9 (C-3), 28.7 (C-4); positive ESI-MS *m/z*: 238 [M−CN]^+^, 265 [M+H]^+^, 287 [M+Na]^+^. The IR, ^1^H-NMR and ^13^C-NMR data are agreement with the literature data [17].

*2-Phenyl-1,2,3,4-tetrahydroisoquinoline-1-carbonitrile* (**24**): white solid; yield: 73%; m.p. 89–90 °C; IR (KBr) *υ*_max_ cm^−1^: 2223 (s, C≡N), 1222, 1206 (C−N); UV (MeOH): λ_max_ (lg *ε*) 245 (4.04) nm; ^1^H-NMR (CDCl_3_) *δ*: 7.39–7.43 (2H, m), 7.31–7.37 (3H, m), 7.27–7.30 (1H, m), 7.14 (1H, s), 7.13 (1H,s), 7.06 (1H, t, *J* = 7.5 Hz), 5.56 (1H, s, H-1), 3.82 (1H, ddd, *J* = 12.5, 6.0, 3.0 Hz, H-3a), 3.53 (1H, ddd, *J* = 12.0, 10.5, 4.0 Hz, H-3b), 3.19 (1H, ddd, *J* = 16.5, 10.5, 6.0 Hz, H-4a), 3.01 (1H, dt, *J* = 16.0, 3.5 Hz, H-4b); ^13^C-NMR (CDCl_3_) *δ*: 148.4 (C-1'), 134.7, 129.7, 129.4, 128.8, 127.1, 126.9, 121.9, 117.8 (C≡N), 117.6, 53.2 (C-1), 44.2 (C-3), 28.6 (C-4); positive ESI-MS *m/z*: 208 [M−CN]^+^. The IR, ^1^H-NMR and ^13^C-NMR data are agreement with the literature data [17].

### 3.3. Acaricidal Activity Assay

The acaricidal activity of compounds **1**–**24** was assayed against *Psoroptes cuniculi* according to our previously reported method [10]. Briefly, adult mites of both sexes were isolated from naturally infected rabbits under a stereoscopic microscope, and placed in 24-well flat-bottomed cell culture plates (20 adult mites/well). The solution of the tested compound with 0.4 mg/mL was prepared in the mixed solution of dimethyl sulfoxide (DMSO), Tween-80 and normal saline (1:1:8, v/v/v). Half a milliliter of the solution was directly added to each well. Three replicates were made for each compound. The same solution without the tested compound was used as untreated control. The solution of ivermectin, a standard acaricide, was used as positive drug control. All plates were placed in separate humidity chambers in saturated humidity conditions at 22 °C. After 24 h, each well was observed under a stereomicroscope and all the motionless mites were stimulated with a needle. Lack of reactions and persistent immobility indicated their death. Mortality was calculated as the following formula and expressed as means ± S.D.:

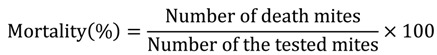
(1)


### 3.4. Acaricidal Toxicity Assay

According to our previously reported method [9], compound **1** with the strongest activity was selected to determine its acaricidal toxicity against *P. cuniculi*. A 2.0 mg/mL stock solution of the tested compound was prepared in a mixture of dimethyl sulfoxide (DMSO), Tween-80 and normal saline (1:1:8, v/v/v), and then diluted with the same mixed solvent to obtain a series of concentrations of 1.8, 1.6, 1.4, 1.2, 1, 0.5, 0.25, 0.125, 0.0625, 0.0313, 0.0157 and 0.0078 mg/mL. The acaricidal activity for each concentration was tested according to the same procedure described above. Each test was performed in triplicate. The mortality of mites for each test was calculated and then corrected by applying Abbott’s formula:

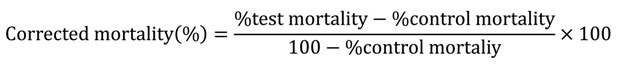
(2)


Probit value of the corrected mortality for each test concentration and the corresponding log[concentration (mg/L)] were used to establish toxicity regression equation with the linear least-square fitting method. The LC_50_ value and its confidence interval at 95% probability were calculated from the toxicity regression equation.

Test solutions of **1** or ivermectin (3 mg/mL) in the same mixed solvent was prepared and used to determine the LT_50_ value. The determination of acaricidal activity of each solution was carried out according to the method described above. The mites in each well were observed under a stereomicroscope every 0.5 h or 1.0 h and the percentage mortality and corrected percentage mortality for each well in each set time was calculated. The tests were performed in triplicate. The corrected percentage mortality at each set time was expressed as means ± S.D. Toxicity regression equation for time effect was established between the probit value of the corrected percentage mortality for each post-treatment time and the corresponding log[post-treatment time (h)], and used to calculate LT_50_.

## 4. Conclusions

In conclusion, we have reported the synthesis of a series of the target compounds and their *in vitro* acaricidal activity against *P. cuniculi*, and also discussed their SAR. All compounds **1**–**24** were found to possess different degrees of activity at 0.4 mg/mL. Among them, **1** displayed the highest activity with an LC_50_ value of 0.2421 μg/mL and an LT_50_ value of 7.79 h, and an average mortality rate of 66.7%, superior to the standard drug ivermectin. The SAR showed that 2'-F, 3'-F, 2'-Cl, 2'-Br and 2'-CF_3_ substituents on the *N*-aromatic ring remarkably enhanced the activity. Thus, the target compounds can be considered a novel class of promising candidates or lead compounds for the development of new tetrahydroisoquinoline acaricidal agents.
